# State-Specific Prevalence of Walking Among Adults with Arthritis — United States, 2011

**Published:** 2013-05-03

**Authors:** Jennifer M. Hootman, Kamil E. Barbour, Kathleen B. Watson, Janet E. Fulton

**Affiliations:** Div of Population Health; Div of Nutrition, Physical Activity, and Obesity, National Center for Chronic Disease Prevention and Health Promotion, CDC

Walking contributes to total physical activity and is an appropriate activity to increase overall physical activity levels among adults with arthritis. Walking also is the most preferred exercise among arthritis patients (1,2) and has been shown to improve arthritis symptoms, physical function, gait speed, and quality of life (3–5). To estimate the distribution of average weekly minutes of walking among adults with arthritis by state and map the prevalence of low amounts of walking (<90 minutes per week) among adults with arthritis, CDC analyzed data from the 2011 Behavioral Risk Factor Surveillance System (BRFSS). This report describes the results of that analysis, which indicated that among adults with arthritis in the 50 states and the District of Columbia (DC), the median prevalence of walking was 53% (range: 44.3%–66.2%) for 0 minutes per week, 13.1% (range: 9.3%–16.2%) for 1–89 minutes per week, 5.3% (range: 3.2%–6.8%) for 90–119 minutes per week, 5.6% (range: 2.6%–8.3%) for 120–149 minutes per week, and 23.2% (range: 16.0%–30.6%) for ≥150 minutes per week. A state median of 66% of adults with arthritis walked <90 minutes per week, ranging from a low of 58.0% in California to a high of 76.2% in Tennessee. The large number of persons with arthritis who are not getting the full benefit of regular walking might benefit from community interventions aimed at increasing access to walking as well as specific programs that offer social support.

BRFSS is a random-digit–dialed telephone survey conducted annually in all 50 states, DC, and U.S. territories. Data collected in 2011 from 50 states and DC (497,967 respondents; 166,417 with arthritis) were used to assess the distribution of average weekly minutes of walking and the prevalence of walking <90 minutes per week among adults with self-reported, doctor-diagnosed arthritis. After excluding responses from respondents with missing data on key variables (e.g., arthritis status and physical activity), the analytic sample size was 153,688 respondents with arthritis. Response rates for BRFSS are calculated using standards set by the American Association of Public Opinion Research response rate formula no. 4.* The response rate is the number of respondents who completed the survey as a proportion of all eligible and likely eligible persons. The 2011 median survey response rate for all states and DC was 53.0%; response rates ranged from 37.4% in California to 66.5% in South Dakota.†

Respondents were classified as having arthritis if they answered “yes” to the question, “Have you ever been told by a doctor or other health professional that you have arthritis, rheumatoid arthritis, gout, lupus, or fibromyalgia?” Respondents who reported they had participated in physical activities or exercise (excluding occupational and transportation activities) in the past month were subsequently asked to recall the frequency, duration, and type of activity for the two activities they did most often. Walking was one of approximately 60 activities listed, and the most common activity reported. For adults who reported walking, the time spent walking per week was calculated by multiplying the frequency (times per week) by duration (minutes per session). Based on the *2008 Physical Activity Guidelines for Americans*, time spent in vigorous-intensity walking (walking is a vigorous-intensity activity for some older adults) was multiplied by two.§

The average number of minutes walked per week was grouped into five categories: 0, 1–89, 90–119, 120–149, and ≥150 minutes per week. Walking minutes were dichotomized to <90 minutes per week and ≥90 minutes per week to assess the state-specific prevalence of low amounts of walking. The 90-minute threshold was based on the minimum amount of weekly walking shown in a randomized controlled trial to lower pain (27% decrease) and improve function (39% increase) among adults with arthritis (5) and the typical amount of walking achieved in the Arthritis Foundation’s Walk With Ease (WWE) program, which is 3 days per week with approximately 30 minutes of total walking time per session (3). Unadjusted prevalence estimates, 95% confidence intervals, medians, and ranges for all 50 states and DC were calculated (Table). Age-adjusted prevalence estimates, categorized by tertiles, also were calculated (Figure). All estimates use sampling weights (raking methodology) to account for the complex sample design, nonresponse, noncoverage, and cellphone-only households; this method of weighting sample BRFSS data is new in 2011; therefore, 2011 estimates should not be compared to estimates from previous years.¶

Among adults with arthritis in the 50 states and the District of Columbia (DC), the median prevalence of walking was 53% (range: 44.3%–66.2%) for 0 minutes per week, 13.1% (range: 9.3%–16.2%) for 1–89 minutes per week, 5.3% (range: 3.2%–6.8%) for 90–119 minutes per week, 5.6% (range: 2.6%–8.3%) for 120–149 minutes per week, and 23.2% (range: 16.0%–30.6%) for ≥150 minutes per week. A median of 66% adults with arthritis walked <90 minutes per week, ranging from a low of 58.0% in California to a high of 76.2% in Tennessee (Table). Among adults with arthritis, eight states had age-adjusted prevalences of walking <90 minutes per week of ≥71.8%, 25 states had prevalences ranging from 65.3 to <71.8% and 18 states had prevalences of <65.3% (Figure).

## Editorial Note

Walking is a low-impact, acceptable, convenient, inexpensive, feasible, and proven physical activity intervention that can help reduce arthritis pain, improve function (3,6), and move persons with arthritis along the continuum of physical activity, getting them closer to meeting the *2008 Physical Activity Guidelines for Americans*. In this study, more than half of adults with arthritis in all 50 states and DC reported no or low (<90 minutes) walking per week. Better access to evidence-based physical activity programs for adults with arthritis will provide increased reach of these programs, which might improve physical activity levels and provide associated health benefits to this population.

The *Guide to Community Preventive Services* recommends both behavioral and social approaches and environmental and policy approaches to increase physical activity.** Individually adapted behavior-change programs that incorporate skills such as goal setting, building social support, and problem solving have been shown to increase time spent in physical activity as well as increase aerobic capacity and energy expenditure. Such programs include the Arthritis Foundation Exercise Program, Senior Services’ EnhanceFitness program, and the Arthritis Foundation’s WWE program.†† Pairing individual, evidence-based physical activity programs with environmental/policy approaches that increase access to physical activity is a feasible way to increase walking among adults with arthritis. For example, worksites that build walking trails or provide walking maps as an environmental approach to increasing employee physical activity might augment their worksite wellness programs by offering an evidence-based program, such as WWE, to employees who desire to increase their walking in a group-lead or self-directed program.

What is already known on this topic?

Walking has been shown to reduce arthritis symptoms and improve physical function, strength, balance, and quality-of-life. Walking is a low-impact, acceptable, convenient, inexpensive, and preferred activity for adults with arthritis and is an appropriate activity to increase overall physical activity.

What is added by this report?

In every state, more than half of adults with arthritis do no or little (<90 minutes) walking per week. Prevalence of walking <90 minutes per week ranged from 58.0% in California to 76.2% in Tennessee. The age-adjusted prevalence of walking <90 minutes per week was ≥71.8% in eight states.

What are the implications for public health practice?

The large number of persons with arthritis who are not getting the full benefit of regular walking might benefit from community interventions aimed at increasing access to walking as well as specific programs that offer social support.

WWE, a 6-week walking program, has been shown to reduce pain and fatigue and increase function, ability, strength, balance, and walking pace among adults with arthritis (3). WWE has two formats, a traditional group-lead version using a trained leader, and a self-directed version where persons can go through the program at their own pace. Typically, WWE groups meet 3 days a week for about an hour, with a maximum walking time of 30–40 minutes per session. Persons with arthritis who walk <90 minutes per week might find that the structure and social support of WWE reduces barriers to walking. The social support of a group walking program also might help improve adherence to a walking program and promote a feeling of safety (6). Currently, CDC funds 12 states to implement evidenced-based physical activity programs in local communities. In the first year of the current 5-year grant cycle, all 12 states offered WWE by partnering with various delivery systems, such as county extension offices, health-care systems and health plans, parks and recreation departments, and organizations serving aging adults.

The findings in this report are subject to at least six limitations. First, all data in BRFSS is based on self-report; therefore, arthritis status and the weekly amount of walking might be misreported. However, the case-finding question used in BRFSS to assess arthritis status has been shown to be sufficiently sensitive and specific for public health surveillance purposes (7). Second, among adults with arthritis, rates of meeting physical activity recommendations via self-reported measures (approximately 30%) are much higher than when activity is objectively measured using motion sensors (13% among men and 8% among women); however, the prevalence of physical inactivity (the low end of the activity spectrum) is similar using both methods (8,9). Third, BRFSS questions do not include transportation or occupational activities that involve walking. Fourth, BRFSS does not assess the severity, location, or type of arthritis, which might affect walking differently. Fifth, because of the sample size, categories (e.g., 1–89 minutes per week) were collapsed so respondents in this category range from being practically inactive to walking an amount that might have important health effects. However, these respondents still are on the low end of the continuum and are good targets for marketing evidenced-based programs. Finally, the 2011 median survey response rate for all states and DC was 53.0% and ranged as low as 37.4% in California; lower response rates can result in response bias.

Most persons with arthritis do no or little walking per week. Effective and safe interventions are available in the community and can assist persons with arthritis to start and maintain a walking program. By coupling environmental and policy strategies to increase access to walking, it might be possible to expand the reach of these effective programs for adults with arthritis.

## Figures and Tables

**FIGURE:**
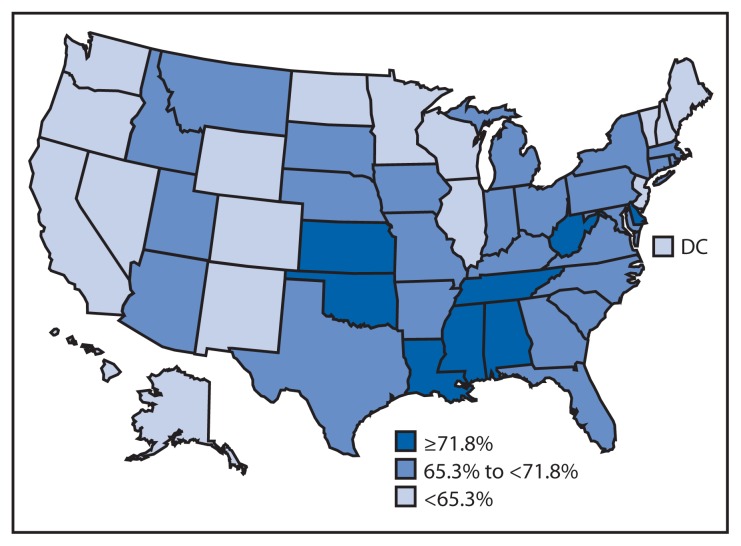
Age-adjusted prevalence of walking <90 minutes per week among adults with arthritis, by state — United States, Behavioral Risk Factor Surveillance System, 2011

**TABLE:** State-specific prevalence of walking among adults with arthritis, by average minutes walked per week — United States, Behavioral Risk Factor Surveillance System, 2011

State	Average minutes walked per week											

	0		1–89		90–119		120–149		≥150		<90	
					
	%	(95% CI)	%	(95% CI)	%	(95% CI)	%	(95% CI)	%	(95% CI)	%	(95% CI)
Alabama	59.7	(57.2–62.2)	14.5	(12.8–16.3)	4.8	( 3.8– 6.1)	4.2	( 3.2– 5.5)	16.8	(15.0–18.6)	74.2	(71.9–76.4)
Alaska	49.8	(45.1–54.5)	10.9	( 8.3–14.2)	4.4	( 2.9– 6.5)	5.5	( 3.5– 8.6)	29.4	(25.2–34.0)	60.7	(56.0–65.3)
Arizona	50.1	(46.2–54.0)	12.8	(10.5–15.7)	5.6	( 4.1– 7.5)	5.1	( 3.7– 7.0)	26.4	(23.1–29.9)	63.0	(59.2–66.6)
Arkansas	59.6	(56.0–63.1)	13.7	(11.3–16.4)	4.2	( 3.0– 6.0)	4.7	( 3.5– 6.2)	17.8	(15.2–20.8)	73.2	(69.9–76.3)
California	44.3	(42.3–46.4)	13.7	(12.3–15.2)	6.6	( 5.5– 7.9)	7.7	( 6.6– 8.9)	27.7	(26.0–29.6)	58.0	(55.9–60.0)
Colorado	46.6	(44.3–48.9)	13.5	(12.0–15.1)	6.6	( 5.6– 7.8)	7.0	( 5.9– 8.4)	26.3	(24.4–28.3)	60.1	(57.8–62.3)
Connecticut	53.3	(50.1–56.4)	12.7	(10.8–14.8)	4.5	( 3.4– 5.9)	5.5	( 4.3– 6.9)	24.1	(21.5–26.9)	65.9	(62.9–68.8)
Delaware	62.5	(58.9–65.9)	10.6	( 8.5–13.1)	4.7	( 3.4– 6.5)	4.1	( 2.9– 5.7)	18.1	(15.5–21.1)	73.1	(69.8–76.1)
District of Columbia	49.4	(45.2–53.7)	9.3	( 7.6–11.3)	4.8	( 3.2– 7.2)	5.8	( 4.4– 7.7)	30.6	(26.9–34.6)	58.7	(54.5–62.8)
Florida	54.1	(51.6–56.5)	11.2	( 9.8–12.8)	4.4	( 3.5– 5.6)	5.7	( 4.5– 7.1)	24.7	(22.6–26.8)	65.2	(62.8–67.6)
Georgia	55.0	(52.3–57.6)	12.3	(10.7–14.0)	5.9	( 4.8– 7.4)	5.8	( 4.7– 7.2)	21.0	(18.9–23.3)	67.3	(64.7–69.7)
Hawaii	50.5	(47.0–54.1)	12.0	( 9.9–14.6)	4.8	( 3.5– 6.4)	6.8	( 5.2– 8.9)	25.9	(22.9–29.1)	62.5	(59.1–65.9)
Idaho	49.7	(46.3–53.2)	14.2	(12.1–16.7)	5.8	( 4.4– 7.7)	8.3	( 6.4–10.6)	22.0	(19.5–24.7)	63.9	(60.6–67.1)
Illinois	50.7	(47.3–54.2)	13.8	(11.6–16.3)	5.3	( 4.1– 6.9)	5.1	( 3.8– 6.7)	25.1	(22.0–28.5)	64.5	(61.0–67.8)
Indiana	55.3	(52.7–57.9)	14.7	(13.0–16.6)	5.6	( 4.5– 7.0)	4.4	( 3.5– 5.4)	20.1	(18.1–22.2)	70.0	(67.5–72.3)
Iowa	51.6	(48.9–54.3)	15.8	(14.0–17.9)	5.4	( 4.3– 6.8)	5.5	( 4.4– 6.8)	21.7	(19.5–24.0)	67.4	(64.9–69.9)
Kansas	56.2	(54.6–57.8)	15.5	(14.4–16.7)	5.0	( 4.4– 5.7)	5.0	( 4.4– 5.7)	18.3	(17.1–19.5)	71.7	(70.3–73.1)
Kentucky	54.7	(52.1–57.2)	14.6	(13.0–16.4)	5.9	( 4.7– 7.3)	5.4	( 4.3– 6.6)	19.5	(17.5–21.6)	69.3	(66.8–71.6)
Louisiana	63.5	(60.9–66.0)	12.2	(10.7–14.0)	3.7	( 2.9– 4.8)	4.6	( 3.6– 5.9)	16.0	(14.1–18.0)	75.7	(73.4–77.9)
Maine	47.5	(45.5–49.4)	12.8	(11.6–14.1)	6.7	( 5.8– 7.8)	6.0	( 5.1– 7.0)	27.0	(25.3–28.8)	60.3	(58.3–62.2)
Maryland	55.0	(52.2–57.8)	13.1	(11.4–15.0)	4.7	( 3.5– 6.1)	6.1	( 4.9– 7.6)	21.1	(19.0–23.5)	68.1	(65.4–70.6)
Massachusetts	53.1	(51.0–55.3)	10.8	( 9.5–12.2)	4.4	( 3.7– 5.2)	5.6	( 4.8– 6.5)	26.2	(24.3–28.1)	63.9	(61.9–65.9)
Michigan	52.1	(49.7–54.5)	14.2	(12.7–16.0)	5.2	( 4.3– 6.3)	6.1	( 5.0– 7.4)	22.4	(20.5–24.4)	66.3	(64.1–68.5)
Minnesota	49.2	(46.8–51.6)	14.8	(13.1–16.6)	5.6	( 4.5– 7.0)	5.2	( 4.3– 6.3)	25.2	(23.2–27.3)	64.0	(61.7–66.3)
Mississippi	59.6	(57.2–62.0)	13.5	(12.0–15.3)	5.9	( 4.9– 7.2)	4.4	( 3.6– 5.4)	16.5	(14.7–18.4)	73.2	(71.0–75.3)
Missouri	56.4	(53.2–59.5)	13.6	(11.5–16.1)	5.0	( 3.8– 6.6)	5.6	( 4.2– 7.3)	19.4	(17.1–22.0)	70.0	(67.0–72.8)
Montana	53.0	(50.3–55.7)	12.5	(10.9–14.3)	4.9	( 3.8– 6.4)	4.9	( 4.0– 6.1)	24.6	(22.4–27.0)	65.5	(62.9–68.1)
Nebraska	53.9	(52.2–55.6)	14.2	(13.1–15.4)	5.5	( 4.7– 6.4)	4.6	( 4.1– 5.3)	21.8	(20.5–23.2)	68.1	(66.5–69.6)
Nevada	54.0	(49.3–58.6)	10.5	( 8.4–13.1)	5.6	( 3.8– 8.1)	5.8	( 4.1– 8.1)	24.2	(20.5–28.3)	64.5	(59.9–68.8)
New Hampshire	51.9	(48.9–54.9)	13.5	(11.5–15.8)	5.2	( 4.1– 6.5)	5.6	( 4.4– 7.1)	23.8	(21.4–26.3)	65.4	(62.6–68.2)
New Jersey	55.0	(52.7–57.3)	10.1	( 8.8–11.6)	4.2	( 3.4– 5.2)	5.4	( 4.4– 6.5)	25.3	(23.4–27.3)	65.1	(62.9–67.3)
New Mexico	50.1	(47.6–52.6)	12.7	(11.2–14.4)	6.2	( 4.9– 7.7)	5.7	( 4.7– 6.9)	25.3	(23.2–27.6)	62.8	(60.3–65.2)
New York	50.6	(47.6–53.6)	13.2	(11.3–15.5)	5.0	( 3.8– 6.5)	6.2	( 4.9– 7.9)	24.9	(22.5–27.5)	63.8	(60.9–66.6)
North Carolina	54.5	(51.8–57.2)	14.6	(12.8–16.6)	6.0	( 4.7– 7.6)	6.5	( 5.4– 7.9)	18.4	(16.3–20.6)	69.1	(66.5–71.6)
North Dakota	53.0	(49.7–56.2)	12.2	(10.4–14.2)	5.9	( 4.7– 7.5)	5.5	( 4.2– 7.2)	23.4	(20.6–26.3)	65.2	(62.0–68.3)
Ohio	54.8	(52.3–57.3)	12.5	(11.0–14.2)	5.4	( 4.4– 6.6)	5.2	( 4.2– 6.3)	22.0	(19.9–24.3)	67.4	(64.9–69.7)
Oklahoma	57.7	(55.3–60.1)	14.7	(13.0–16.5)	4.6	( 3.7– 5.7)	4.7	( 3.8– 5.8)	18.4	(16.6–20.3)	72.4	(70.2–74.5)
Oregon	47.6	(44.7–50.5)	13.2	(11.3–15.4)	5.9	( 4.7– 7.5)	7.1	( 5.8– 8.6)	26.1	(23.8–28.6)	60.9	(58.1–63.6)
Pennsylvania	52.2	(49.9–54.5)	12.4	(10.9–14.0)	5.8	( 4.8– 7.1)	6.2	( 5.2– 7.3)	23.4	(21.6–25.3)	64.6	(62.4–66.7)
Rhode Island	52.6	(49.8–55.4)	12.9	(11.2–14.8)	4.5	( 3.6– 5.8)	6.2	( 5.0– 7.6)	23.8	(21.4–26.3)	65.5	(62.8–68.2)
South Carolina	52.9	(50.6–55.3)	14.5	(13.0–16.2)	5.4	( 4.5– 6.5)	6.2	( 5.0– 7.6)	20.9	(19.1–22.8)	67.5	(65.2–69.7)
South Dakota	52.1	(47.9–56.1)	16.2	(13.6–19.2)	5.7	( 3.9– 8.2)	5.7	( 4.1– 8.1)	20.3	(17.2–23.9)	68.3	(64.2–72.0)
Tennessee	66.2	(61.7–70.4)	10.0	( 8.0–12.5)	3.6	( 2.5– 5.1)	2.6	( 1.9– 3.6)	17.5	(14.1–21.6)	76.2	(72.0–79.9)
Texas	54.2	(51.3–56.9)	15.0	(12.9–17.2)	5.4	( 4.3– 6.7)	5.5	( 4.3– 7.0)	20.0	(18.0–22.1)	69.1	(66.5–71.6)
Utah	49.0	(46.6–51.4)	14.9	(13.3–16.7)	6.1	( 5.1– 7.4)	6.7	( 5.7– 8.0)	23.2	(21.3–25.2)	63.9	(61.7–66.2)
Vermont	49.5	(46.7–52.2)	12.2	(10.6–14.0)	6.1	( 5.0– 7.5)	6.3	( 5.1– 7.7)	26.0	(23.6–28.5)	61.6	(58.9–64.3)
Virginia	58.4	(55.2–61.5)	12.2	(10.3–14.4)	4.5	( 3.4– 6.0)	6.3	( 4.8– 8.1)	18.7	(16.4–21.1)	70.6	(67.7–73.4)
Washington	46.8	(44.3–49.3)	14.2	(12.6–15.9)	5.3	( 4.3– 6.6)	6.5	( 5.4– 7.8)	27.3	(25.1–29.5)	61.0	(58.5–63.3)
West Virginia	62.2	(59.6–64.7)	12.1	(10.5–13.9)	3.2	( 2.4– 4.2)	3.6	( 2.8– 4.6)	18.9	(16.9–21.1)	74.3	(72.0–76.6)
Wisconsin	47.1	(43.1–51.1)	12.2	( 9.9–14.9)	6.8	( 4.6– 9.9)	5.6	( 4.3– 7.3)	28.3	(24.9–32.0)	59.3	(55.3–63.2)
Wyoming	49.8	(46.2–53.3)	12.1	(10.1–14.5)	4.8	( 3.5– 6.6)	5.0	( 3.7– 6.6)	28.3	(25.1–31.8)	61.9	(58.3–65.4)
*Median*	*53.0*	*(51.6–54.2)*	*13.1*	*(12.5–13.7)*	*5.3*	*(4.9–5.6)*	*5.6*	*(5.4–5.8)*	*23.2*	*(21.1–24.7)*	*65.5*	*(64.5–67.5)*
*Range*	*44.3–66.2*		*9.3–16.2*		*3.2–6.8*		*2.6–8.3*		*16.0–30.6*		*58.0–76.2*	

**Abbreviation:** CI = confidence interval.

## Footnotes

* Response rate definitions http://www.aapor.org/standard_definitions2.htm.

† *2011 Behavioral Risk Factor Surveillance Survey Summary Data Quality Report* available at http://www.cdc.gov/brfss/pdf/2011_summary_data_quality_report.pdf.

§ *2008 Physical Activity Guidelines for Americans* available at http://www.health.gov/paguidelines.

¶ 2011 BRFSS overview available at http://www.cdc.gov/brfss/annual_data/annual_2011.htm.

** *Guide to Community Preventive Services* available at http://www.thecommunityguide.org/pa/index.html.

†† *Arthritis Appropriate Physical Activity and Self-Management Education Interventions: A Compendium of Implementation Information* available at http://www.cdc.gov/arthritis/interventions/marketing-support/compendium/docs/pdf/compendium-2012.pdf.
